# Association of IDH1 Mutation and MGMT Promoter Methylation with Clinicopathological Parameters in an Ethnically Diverse Population of Adults with Gliomas in England

**DOI:** 10.3390/biomedicines12122732

**Published:** 2024-11-29

**Authors:** Hiba A. Wanis, Henrik Møller, Keyoumars Ashkan, Elizabeth A. Davies

**Affiliations:** 1 Cancer Epidemiology and Cancer Services Research, Centre for Cancer, Society & Public Health, Bermondsey Wing, King’s College London, 3rd Floor, Guy’s Hospital, Great Maze Pond, London SE1 9RT, UK; 2 Danish Centre for Health Services Research, Aalborg University, 9220 Aalborg, Denmark; 3 Department of Neurosurgery, King’s College Hospital NHS Trust, Denmark Hill, London SE5 9RS, UK

**Keywords:** brain tumours, IDH1 mutation, MGMT promoter methylation, odds ratio, clinicopathological parameters, health inequalities

## Abstract

**Background:** Molecular profiles can predict which patients will respond to current standard treatment and new targeted therapy regimens. Using data from a highly diverse population of approximately three million in Southeast London and Kent, this study aims to evaluate the prevalence of IDH1 mutation and MGMT promoter methylation in the gliomas diagnosed in adult patients and to explore correlations with patients’ demographic and clinicopathological characteristics. **Methods:** Anonymised data on 749 adult patients diagnosed with a glioma in 2015–2019 at King’s College Hospital were extracted. Univariable and multivariable logistic regressions were used to estimate odds ratios (ORs) for expressing IDH1 mutation and MGMT promoter methylation, based on each patient’s age, sex, ethnicity, histology, tumour location and extent of resection. The Kaplan–Meier method was used to estimate the overall survival functions. **Results:** A total of 19.5% of cases were IDH1-mutated. Being 39 years and younger (OR 5.48, 95% CI 3.17–9.47), from Asian/Asian British background (OR 3.68, 95% CI 1.05–12.97), having MGMT methylation (OR 15.92, 95% CI 7.30–34.75), an oligodendroglioma diagnosis (OR 7.45, 95% CI 2.90–19.13) and receiving a gross total/total microscopic resection (OR 1.95, 95% CI 1.24–3.08) were each univariately correlated with IDH1 mutation. MGMT methylation association persisted on adjustment (OR 14.13, 95% CI 3.88–51.43). MGMT promoter methylation was seen in 54.3% of gliomas. In the univariate adjusted ORs, being younger than 39 years (OR 2.56, 95% CI 1.48–4.43), female (OR 1.52, 95% CI 1.11–2.08), having IDH1 mutation (OR 15.92, 95% CI 7.30–34.75) and an oligodendroglioma diagnosis (OR 6.20, 95% CI 1.33–28.88) were associated with MGMT methylation. Being female (OR 1.75, 95% CI 1.22–2.51) and having an IDH1 mutation (OR 15.54, 95% CI 4.73–51.05) persisted after adjustment for age, sex, ethnicity, histology, tumour location and extent of resection. IDH1 mutant and MGMT methylated gliomas were associated with frontal lobe location. Survival analysis showed that patients with both IDH1 mutation and MGMT methylation had significantly better survival than those with either molecular marker alone. Over a 3-year period, women with unmethylated MGMT promoters generally had better survival than men with unmethylated MGMT. **Conclusion:** This study showed that the molecular markers of IDH1 mutation and MGMT promoter methylation were associated with age, sex, Asian/Asian British ethnic group, tumour histology, anatomical location and extent of resection. This study has demonstrated the importance of assessing glioma molecular markers in the clinical setting and the need to stratify patients according to their clinicopathological characteristics.

## 1. Introduction

The classification term glioma includes a broad range of tumours originating from the glial brain cells. Gliomas occur mostly in adults, most frequently in the frontal lobe, and have a higher incidence in males [1,2]. The most common glioma subtypes are glioblastoma, followed by grade II and III astrocytoma and oligodendroglioma [2]. Their diagnosis, treatment and prognosis depend on several key variables, including the patient’s age, the tumour subtype and its location, with new findings indicating the significance of certain molecular biomarkers [1]. Understanding the molecular characteristics of glioma has improved our knowledge of disease progression and response to treatment and opened up new opportunities for prognostication [3]. With the introduction of the 2016 and, more recently, the 2021 WHO Central Nervous System (CNS) tumour classification, both molecular and histopathologic features have now been incorporated into classification schemes. By integrating genetic and epigenetic information to define tumour entities, these molecular biomarkers may give patients a more precise diagnosis and prognosis of their tumours [4,5].

The main biomarkers that are now routinely tested for in clinical practice are isocitrate dehydrogenase (IDH) gene mutations (mainly IDH1), O6-methylguanine-DNA methyltransferase (MGMT) promoter methylation and the co-deletion of 1p/19q. An IDH1-R132H point mutated enzyme (mutation in IDH1 at R132) is seen in 80–90% of grade II-III gliomas. Much less commonly, in less than 1% of IDH mutant gliomas, is IDH2 (mutation at R172). IDH1/2 are key enzymes in the tricarboxylic cycle and glutamine metabolism. Although histologically indistinguishable, primary and secondary glioblastomas are genetically and epigenetically different. Mutations in IDH1 and, less frequently, IDH2 now define a secondary glioblastoma (low-grade gliomas that eventually underwent a malignant transformation) [3,6], whereas the IDH1 wild-type is considered to be a primary glioblastoma that arose as a higher-grade tumour.

Equally important to the biomarker IDH1 mutation is MGMT gene silencing by methylation, which is found in about 50% of all newly diagnosed glioblastoma [7]. MGMT is a DNA repair enzyme that removes alkyl groups from the O6 position of guanine nucleotide, which is the critical site modified by alkylating chemotherapeutics [8]. This repair activity allows MGMT to reverse the damage induced by temozolomide (TMZ). Approximately 40% of gliomas exhibit MGMT methylation, leading to decreased MGMT expression i.e., decreased DNA repair and therefore to an enhanced sensitivity to TMZ, which significantly prolongs survival compared to patients without the methylation of this site [8]. Thus, IDH1 mutations and MGMT methylation are recognised as independent prognostic factors affecting overall survival [8,9].

The association between molecular biomarkers and progression-free survival and overall survival has been well investigated, particularly for glioblastoma. This paper concentrates on the IDH1 mutation and MGMT promoter methylation and their associations with various clinicopathological characteristics in low-grade and high-grade glioma. The patient population is those living in Southeast London and Kent and seen at King’s College Hospital.

## 2. Material and Methods

### 2.1. Study Population

Data on all patients aged 18 years or over, diagnosed with a glioma between 1 January 2015 and 31 December 2019 and discussed in the Multi-Disciplinary Team meeting (MDT) at King’s College Hospital NHS Trust were extracted. These data included information on demographic characteristics, tumour type, referral and surgery details.

### 2.2. Selection of Cases

Patients were given a histologically confirmed diagnosis using ICD-10 and morphology codes according to the WHO classification of CNS tumours. The brain tumour morphological subtypes considered in this analysis were glioblastoma (morphology codes: 9440–9442, 9445), astrocytoma that excludes glioblastoma (morphology codes: 9381, 9384, 9400–9411, 9420–9421, 9424, 9425) and oligodendroglioma (morphology codes: 9450–9451). The 2016 WHO classification was used to categorise tumour morphology. The WHO CNS classification was updated in 2021, but these changes have minimal effect on the analysis of the extracted data. Data were then cross-checked against the patients’ MDT reports and neuropathology records for accuracy and validity.

These records include self-assigned ethnicity data provided by patients upon admission to King’s College Hospital Neurosciences Department. Due to the small numbers of patients in some of the ethnicity categories the following groups were used for analysis: White British, Asian/Asian British, Black/Black British, Mixed Ethnic Groups, Any Other White, Any Other Ethnic Group and Unknown/Not Stated.

The Neuropathology Department at King’s College Hospital carries out nearly 400 molecular pathology tests per year [10]. For all molecular pathology tests, ten 4–5 µm thick unstained formalin-fixed paraffin-embedded (FFPE) tissue sections are required. DNA testing of MGMT promoter methylation status and the sequencing of IDH1 and IDH2 are carried out using pyrosequencing. Co-deletion of 1p/19q test is performed using fluorescence in situ hybridisation (FISH). The MGMT methylation status is reported as the percentage of methylation (<5% = unmethylated, 5–10% = borderline methylated and >10% = methylated). Following a similar survival outcome between methylated and borderline methylated, and due to the low number of tumours in the borderline group, these two categorisations of methylation were combined to form one group: ‘MGMT-methylated’.

The initial extraction comprised 770 records. We then cleaned the data to exclude duplicated cases, metastatic tumours (18 cases) as well as those originating in the spine (2 cases) from the analysis. Survival time was calculated from the date of diagnosis until date of death with a period of observation of up to one year. Data on one patient who died before their date of diagnosis were removed. In order to retain those who died on their date of diagnosis, we added half a day to their survival time (4 patients were retained). The final study population included 749 patients.

### 2.3. Data Analysis

Firstly, we began by examining the distribution of patients by person-level characteristics (age, sex, ethnicity and performance status at referral), tumour morphology, tumour WHO grade, tumour location, molecular characteristics (IDH1 mutation, MGMT promoter methylation and 1p19q co-deletion) and extent of resection. The Kaplan–Meier (KM) method was used to estimate the overall survival functions. Data for patients who did not experience death were censored on the study end date (30 July 2021) or the last MDT date available in the database, whichever occurred first. Logistic regression was used to estimate odds ratios (OR) (and their 95% confidence intervals (CI)) for IDH1 mutation and then for MGMT promoter methylation and their associations with age, sex, ethnicity, tumour location, tumour histology and extent of resection. ORs were adjusted sequentially for age, sex, performance status at referral, molecular biomarkers, tumour histology, tumour location and extent of resection. χ2 tests were performed to estimate the *p*-values for trend and heterogeneity, excluding unknown categories. All analyses were performed using Stata Software, version 16 (StataCorp, College Station, TX, USA).

### 2.4. Ethical Approval

This study involved anonymised information about the patients and received Health Research Authority (HRA) approval (IRAS Number 228103) and was approved by the local Neurosciences Research Advisory Group Meeting at King’s College Hospital NHS Foundation Trust.

## 3. Results

Table 1 summarises the characteristics of glioma patients, their tumour anatomical locations and molecular profiles. Most patients diagnosed with a glioma were male (60.5%), and approximately half were aged 50–69 years. The majority of cases were high-grade glioma, i.e., WHO grade III/IV (89.7%), and of those, 76.1% were classified as a glioblastoma. The most frequent location within the brain was the frontal lobe (31.6%). Further breakdown of glioma histology by molecular characteristics is shown in Table 2. The proportion of tumours tested for IDH1 mutations and MGMT promoter methylation were high (97.9% and 92.3%, respectively). For a confirmed oligodendroglioma diagnosis, testing for 1p/19q co-deletion along with IDH1 mutation is required, and hence, molecular information for both biomarkers is available.

### 3.1. Significance of IDH1 Mutation

Table 3 shows that in the unadjusted analyses, each of the covariates except for sex was correlated with having an IDH1 mutation. There were higher odds of expressing IDH1 mutation for younger patients (OR 5.48, 95% CI 3.17–9.47, in the ≤39 years group), those from an Asian/Asian British background (OR 3.68, 95% CI 1.05–12.97) or those with MGMT promoter methylation (OR 15.92, 95% CI 7.30–34.75) or an oligodendroglioma diagnosis (OR 7.45, 95% CI 2.90–19.13) and those receiving a gross total/total microscopic resection (OR 1.95, 95% CI 1.24–3.08). Having a glioblastoma diagnosis was less likely to be associated with IDH1 mutation (OR 0.02, 95% CI 0.01–0.04). In the adjusted model, the associations with MGMT methylated promoter (OR 14.13, 95% CI 3.88–51.43), receiving a gross total/total microscopic resection (OR 2.73, 95% CI 1.01–7.33) and a glioblastoma diagnosis (OR 0.03, 95% CI 0.01–0.09) persisted. IDH1 mutation was also associated with frontal lobe tumour location. Table 3 shows the odds ratios for IDH1 mutation in glioma cancer patients diagnosed at King’s College Hospital between 2015 and 2019.

### 3.2. Significance of MGMT Promoter Methylation

Table 4 shows the factors correlated with the MGMT promoter methylation group (the combined group of methylation and borderline methylation). In the unadjusted ORs, each of the covariates was associated with MGMT promoter methylation except for ethnicity and extent of resection. The methylation of the MGMT promoter was correlated with younger patients who were aged less than 39 years (OR 2.56, 95% CI 1.48–4.43), female (OR 1.52, 95% CI 1.11–2.08) or had an IDH1 mutation (OR 15.92, 95% CI 7.30–34.75) and an oligodendroglioma diagnosis (OR 6.20, 95% CI 1.33–28.88). Being female (OR 1.75, 95% CI 1.22–2.51) and having an IDH1 mutation (OR 15.54, 95% CI 4.73–51.05) persisted on adjustment. There are higher odds in older patients (60–69 years) (OR 1.76, 95% CI 1.11–2.78) with this epigenetic alteration. MGMT promoter methylation was also associated with frontal lobe tumour location.

### 3.3. Survival Analysis

**Figure 1:**
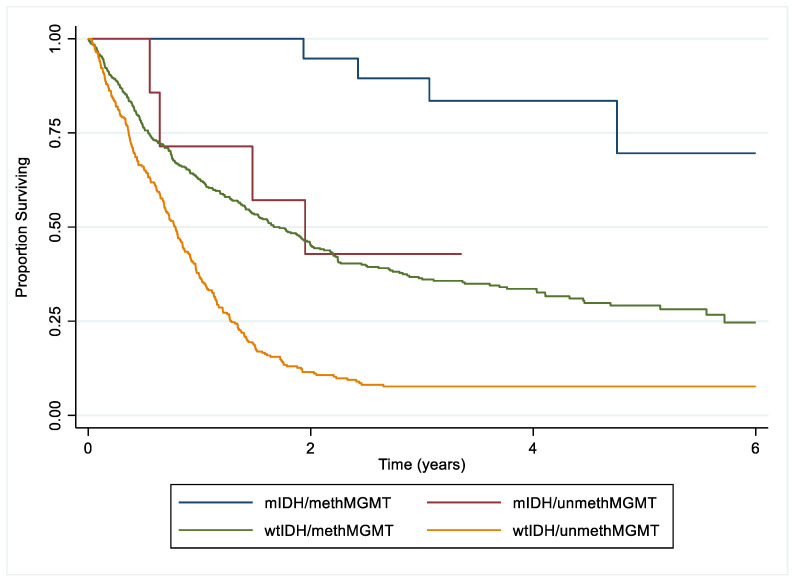
Kaplan–Meier survival curves of 749 King’s College Hospital glioma patients comparing IDH1 wild-type and unmethylated MGMT promoter (wtIDH/unmethMGMT); IDH1 wild-type and methylated MGMT promoter (wtIDH/methMGMT); IDH1 mutation and unmethylated MGMT promoter (mIDH/unmethMGMT); IDH1 mutation and methylated MGMT promoter (mIDH/methMGMT) (*p* < 0.001). *p*-values were calculated by the log-rank test.

The results of this study have shown that there is an association between IDH1 mutation and MGMT promoter methylation. The combined effect of these two important biomarkers on overall survival is observed in the Kaplan–Meier survival curve (Figure 1). Glioma patients with both the IDH1 mutation and MGMT methylation (3.0% of all patients) had the best overall survival, followed by patients with an IDH1 mutation and unmethylated MGMT promoter (1.0%), then by patients with MGMT promoter methylation and IDH1 wildtype (55.4%). Those without an IDH1 mutation or MGMT methylation (40.6%) had the worst overall survival. Patients with only an IDH1 mutation did not have significantly different survival from patients with only MGMT promoter methylation.

**Figure 2:**
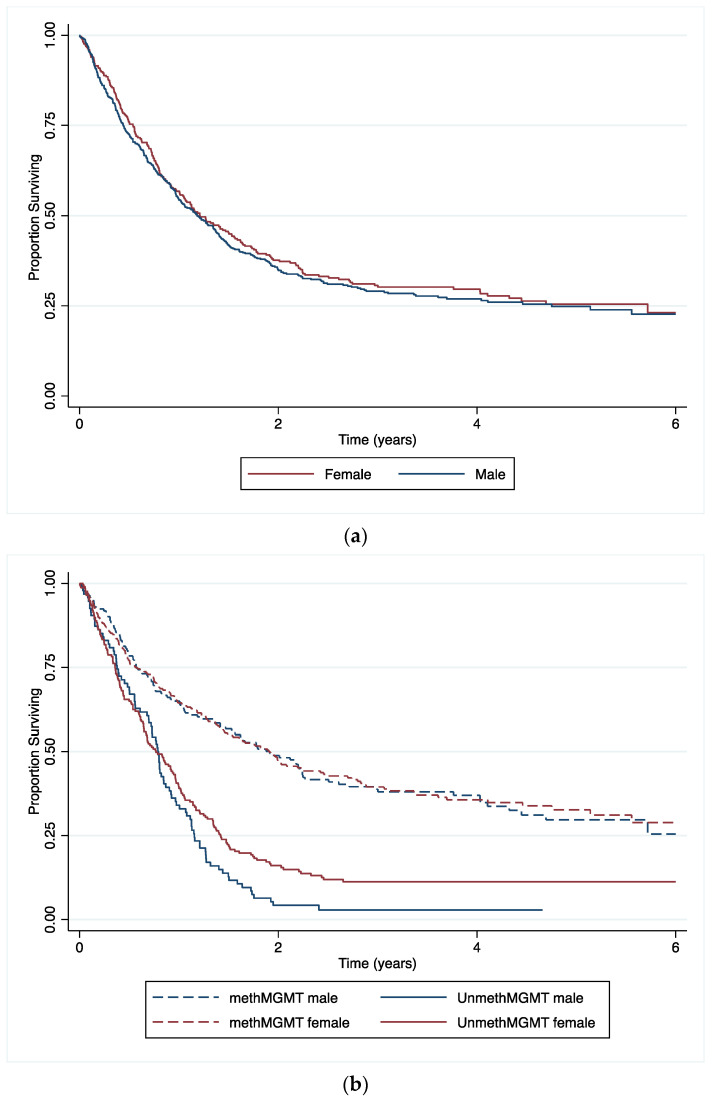
Kaplan–Meier survival curves of the King’s College Hospital glioma patient cohort (**a**) for males (n = 453) vs. females (n = 296) (*p* = 0.521). (**b**) according to MGMT promoter methylation status for males (methMGMT = 171; unmethMGMT = 94) vs. females (methMGMT = 236; unmethMGMT = 197) (*p* < 0.001). *p*-values were calculated by the log-rank test.

Figure 2a shows the survival by sex for the patient cohort (*p* = 0.5215) and when it is stratified by MGMT methylation status as presented in Figure 2b. For the unmethylated MGMT promoter group, the survival for women was still better than for the unmethylated group of men over a longer period of time.

## 4. Discussion

### 4.1. Main Findings

Using information extracted from a highly diverse population of around three million in Southeast London and Kent, we investigated data on 749 adult patients diagnosed with a glioma at King’s College Hospital between 2015 and 2019. This study’s aim was to explore factors associated with IDH1 mutation and/or MGMT promoter methylation. We found that 19.5% of cases were IDH1-mutated. Being young (OR 5.48, 95% CI 3.17–9.47), from an Asian/Asian British background (OR 3.68, 95% CI 1.05–12.97), having MGMT promoter methylation (OR 15.92, 95% CI 7.30–34.75), an oligodendroglioma diagnosis (OR 7.45, 95% CI 2.90–19.13) and receiving a gross total/total microscopic resection (OR 1.95, 95% CI 1.24–3.08) were univariately correlated with IDH1 mutation. MGMT methylation association persisted on adjustment (OR 14.13, 95% CI 3.88–51.43). MGMT promoter methylation was seen in 54.3% of gliomas. In the univariate adjusted ORs, being young (OR 2.56, 95% CI 1.48–4.43), being female (OR 1.52, 95% CI 1.11–2.08), having IDH1 mutation (OR 15.92, 95% CI 7.30–34.75) and an oligodendroglioma diagnosis (OR 6.20, 95% CI 1.33–28.88) were associated with MGMT methylation. Being female (OR 1.75, 95% CI 1.22–2.51) and having IDH1 mutation (OR 15.54, 95% CI 4.73–51.05) persisted after adjustment. IDH1 mutant and MGMT promoter methylated gliomas were also associated with a frontal lobe location. Survival analysis showed that patients with both IDH1 mutation and MGMT promoter methylation had significantly better survival than those with either molecular biomarker. Over a longer period, women with unmethylated MGMT promoters generally had better survival than men with unmethylated promoters.

### 4.2. Comparison to Other Findings

There have been several population-based studies investigating molecular biomarkers and glioma subtypes; however, only a few have considered the associations of such biomarkers and patients’ clinicopathological characteristics that could play an important role in determining prognosis.

One study by Pandith et al. (2020) evaluated the associations of IDH1/IDH2 and MGMT promoter methylation with clinical variables in glioma patients [11]. They reported IDH1 mutation as being also associated with oligodendrogliomas and astrocytomas (*p* = 0.005) and being more frequent in males (*p* = 0.0002) but not significant among age groups [11]. IDH1 mutation was correlated with MGMT promoter methylation (OR = 3.6, 95% CI 1.1–11.1). Multivariate survival analysis showed better survival rates in patients with MGMT methylation following adjustment for age, sex, grade and IDH1/2 mutation. By comparison, our new results showed an association between IDH1 mutation and age. These findings can be compared to previous work by Molenaar et al. (2014) who presented an independent two-gene predictive and prognostic factor for glioblastoma based on IDH1 mutation and MGMT methylation status [12]. Patients with IDH1 mutation as well as MGMT methylation had the longest survival compared to either IDH1 mutations or MGMT methylation alone. Here, similar results are found where the combination of IDH1 mutation and MGMT promoter methylation predicts an improved survival compared to the remaining three combinations, with the worst prognostic group being those with wild-type IDH1 plus an unmethylated promoter. A study by Aurora et al. (2018) has also reported that the MGMT methylation group did not show a statistically significant correlation with age, sex or tumour location, but it was associated with IDH1 mutation (OR 4.3 CI 1.3–13.8) [13].

### 4.3. Interpretation and Implications

Low-grade glioma typically occurs more commonly in younger people than high-grade glioma [14]. In glioblastoma, IDH1 mutations are more common in younger patients and in those diagnosed with a secondary glioblastoma [15]. Our study showed that younger patients are more likely to be diagnosed with an IDH1-mutated glioma than with a wild-type form and that this mutation is rarely found in the elderly patient population. Previous work has shown that patients with grade II–IV gliomas harbouring IDH1 or IDH2 mutation had significantly longer overall survival than those without IDH mutation [16]. These findings are therefore consistent with previous studies that reveal the prognostic importance of IDH mutation independently of other known prognostic factors such as age, grade and MGMT methylation status [16]. In the analysis undertaken for MGMT methylation status in this study, patients under the age of 39 years were more likely to have MGMT methylated promoter in the unadjusted model. However, after sequentially adjusting for tumour characteristics as well as surgery, there was a stronger correlation with this among older patients. It would be of interest to investigate the association between older patients and increased odds of MGMT methylation, in IDH1 mutant astrocytomas.

Glioblastoma occurs more frequently in the male population (male-to-female ratio 1.6:1) [17], with less of a sex difference being found for low-grade astrocytomas [18,19]. Reports show that being female is also associated with a better outcome [20,21]; thus, sex differences in glioma progression have received more interest, particularly at a molecular level, and there is accumulating evidence that gliomas display sex-specific methylation patterns [22]. In glioblastoma, MGMT promoter methylation is common in females, with a subsequent better outcome after treatment with TMZ. This study has confirmed previous findings correlating females with methylated MGMT promoter status. The results also show that even without MGMT methylation, females still have a better survival than males. Taken together, our data illustrates the need for stratification by sex in clinical cohorts of patients with gliomas, where an unequal frequency of gliomas between males and females may disguise sex-specific association with prognosis.

The multivariate analysis found no significant associations between either IDH1 mutation or MGMT methylation and anatomic location in the brain. However, in the unadjusted analysis, both IDH1 mutation and MGMT promoter methylation were associated with frontal lobe gliomas. In other studies, the MGMT methylation was seen predominately in the parietal and occipital lobes [23], temporal lobe [24] or independent from tumour location [25]. Unmethylated cases were commonly located in the temporal lobe [13,23]. Paldor et al. (2016) suggested that glioblastomas arising in the frontal lobe are more prone to exhibiting IDH mutation (*p* = 0.006) and MGMT methylation (*p* = 0.005) than glioblastomas arising in other lobes [26].

In this study, the incidence of IDH1 mutation and MGMT promoter methylation were comparable to figures found globally. A study by Ang et al. (2020) based on a Southeast Asian cohort, includes an ethnically diverse population of Chinese, Malays and Indians. Indians were found to have the highest incidence of positive MGMT methylation and IDH1 mutation results (50% and 30%, respectively) [27]. Our previous work (Wanis et al. 2023) found better survival for those of Indian background after adjusting for patient characteristics, tumour morphology and route to diagnosis and treatment, compared to British White people [28]. Here in this study, the results have shown that individuals from the Asian/British Asian Ethnic Group have higher odds of DH1 mutation compared with the White British Group in the univariate analysis. We suggested a possible explanation for this is that there might be a correlation between those from an Indian background and having an IDH1 mutation [28]. In one study based in India, Indian patients with glioma were revealed as more likely to be diagnosed with a low-grade glioma and to have a higher prevalence of IDH1 mutation, giving them an improved outcome [29]. Other Indian studies also showed a greater IDH1 mutation prevalence reaching as high as 84% of Indian patients tested [30]—assuming that all aggressive tumours are being given a confirmed diagnosis. To address this interesting question, larger and more detailed analyses are needed to determine if there are genetic or other relationships between British-based, Indian-based ethnic groups and IDH1 mutation prevalence.

It has become increasingly evident that IDH mutation status accounts for much of the prognostic knowledge previously characterised by histological subtype and grade [31]. As replicated in our study, most glioblastomas are IDH wild-type, and most lower grade diffuse gliomas are IDH-mutant. IDH mutant status therefore offers the basis for an alternative method of classification. Since the 2016 WHO CNS tumour classification, the presence of IDH mutation is required to diagnose oligodendroglioma, along with 1p/19q co-deletion [4]. While nearly all oligodendrogliomas are MGMT-methylated, this routine testing might not be necessary. IDH mutation increases overall genomic methylation and is associated with MGMT promoter methylation. Additionally, in one study, the MGMT methylation was also seen in nearly all IDH-mutated astrocytomas and oligodendrogliomas [32]. However, this has also been questioned [12], and a detailed analysis is needed to obtain a better understanding of these associations, particularly for grade II–III astrocytomas and oligodendrogliomas.

The methylation of the MGMT promoter was apparent in approximately 50% of newly diagnosed glioblastomas in a few studies as well as this [33,34,35]. Studies have also demonstrated that patients with MGMT promoter methylated glioblastomas have a significantly longer overall survival regardless of treatment [24]. Associations between IDH1 mutation, MGMT promoter methylation and survival outcomes have also been analysed [36]. MGMT methylation is also associated with IDH1 mutant tumours and therefore is more common in secondary than in primary glioblastoma (75% vs. 36% respectively) [23,37]. This molecular biomarker is associated with better response to TMZ as well as radiotherapy [38], and its prognostic and predictive significance in glioblastoma patients remains irrespective of treatment choices. Regarding the extent of resection, studies have shown no benefit of gross total resection and MGMT promoter methylation [39,40,41]. In our study, there was also no association between MGMT promoter methylation and the extent of resection.

Surgery is the first crucial phase in classifying and managing gliomas. Neuro-oncologists and neurosurgeons now face the challenge of incorporating these new molecular biomarkers into the treatment of glioma patients, as evidence for using them in clinical decision-making is evolving. A review by Li et al. (2020) highlighted the potential effect of molecular biomarkers on informing surgical resection decisions for diffuse astrocytomas [42]. Surgical decisions are usually made as a result of a biopsy’s pathology and molecular analyses; however, real-time molecular analyses as tumours are resected could inform surgical decision-making, balancing potential harm from surgical procedures against benefit. It could be determined from this work that IDH1-mutated gliomas are less diffuse and therefore easier to resect than IDH1 wild-type gliomas, and hence, a correlation between IDH1-mutated gliomas and gross total/total microscopic resection type was found. The use of additional treatment data to explore whether the IDH mutation also influences survival in patients receiving radiation or chemotherapy following resection would add useful information. In the NOA-04 phase III trial, comparing radiotherapy with alkylating chemotherapy as an initial treatment of grade III glioma showed an association of IDH mutation with improved overall survival in both treatment groups approximately equally [43]. Conclusions from our study also included that IDH1 mutation is a new positive prognostic factor in anaplastic gliomas, with the extent of resection being an important prognosticator [44]. MGMT promoter methylation was also associated with prolonged PFS in the chemotherapy and radiotherapy arm [43].

### 4.4. Strengths and Limitations

The hospital data used in our study has both strengths and limitations. In addition to the data covering a highly diverse population in Kent and Southeast London of approximately 3 million [45], robustness following an audit of the database was a major strength. Compared to the National Cancer Registry—NDRS, this MDT database has much more extensive information on the extent of resection and surgery type. One major limitation, however, is the absence of linked chemotherapy and radiotherapy data, which could potentially be obtained by data linkage to other hospital treatment databases. Previous work has also considered how molecular markers may be used in clinical decision-making for recurrent surgery for high-grade gliomas particularly for unmethylated MGMT promoter and wildtype IDH1 gliomas—since no other treatment option has demonstrated a survival benefit for these subgroups. Even though this database does not gather information on recurrences, these could be traced using medical history and MDT reports. Tracing could also be used to distinguish between primary and secondary glioblastoma with their differing genetic alteration profiles and prognostic significance. For example, secondary glioblastomas have a far better prognosis with longer median survivals than IDH wild-type primary glioblastomas [6]. Ideally, these data should include a longer duration of follow-up for mortality to consider long-term survival, particularly for low-grade astrocytomas. Furthermore, the study population is limited by relatively small sample sizes for some subgroups, particularly for ethnicity and tumour histology. These variables were broadly grouped making it difficult to draw significant conclusions for smaller populations and limiting the comparison of molecular alterations between defined ethnic groups and astrocytoma specified by WHO grade. Having more data could also give insight into rarer tumour histologies and locations within the brain. Finally, due to the significant genetic heterogeneity within each tumour subtype, the analysis would be strengthened if other genetic alterations, for example, p53 and ATRX mutation, were considered, as these might play a role in IDH1 mutation associations. The absence of the ATRX protein and the abundance of p53 protein are required for classifying low-grade astrocytomas [46,47,48]. Other emerging molecular biomarkers that are more specific to younger adults would also be of interest to investigate such as H3G34 mutation and histone H3K27M mutation. In addition to considering detailed molecular data, a larger study sample will therefore maximise the clinical relevance of these findings if the updated 2021 WHO CNS classifications are implemented (e.g., reassigned IDH-mutated glioblastoma to IDH-mutant astrocytoma).

## 5. Conclusions

Our study has demonstrated the importance of assessing glioma molecular biomarkers in the clinical setting, and the need to stratify patients according to their clinicopathological characteristics. Of the glioma patients diagnosed and treated at King’s College Hospital, 19.5% had IDH1-mutated tumours and 54.3% were MGMT promoter methylated. This analysis showed these molecular biomarkers being associated with sex, age, ethnicity, tumour histology, anatomical location and extent of resection. The results of these analyses substantiate and support recent efforts to increase the extent of resection in gliomas particularly for those tumours with IDH1 mutation and for considering sex-specific associations. Such reports may encourage clinical trials to consider including molecular biomarker characteristics either when allocating patients to trial arms or by stratifying the analysis. These findings can also assist the selection of treatment options for patients based on their clinicopathological characteristics.

**Simple Summary:** Using hospital data that cover a highly diverse population of around three million in Southeast London and Kent, this study showed the associations of the glioma molecular markers of IDH1 mutation and MGMT promoter methylation with a patient’s age, sex, ethnicity, tumour histology, brain location and extent of resection. These results should galvanise efforts to better understand the molecular characteristics of gliomas in order to detect these tumours earlier and maximise treatment management. This study also demonstrated the importance of assessing the translation of these molecular markers in the clinical setting. Not only do they inform prognosis, but they are also predictive in helping the selection of treatment options for glioma patients based on their clinicopathological characteristics. 

## Figures and Tables

**Table 1:** Patient and tumour characteristics of 749 patients diagnosed with a glioma during 2015–2019.

	Variables	Groups	No. of Patients	(%)
**Patient characteristics**	**Sex**	Female	296	(39.5)
		Male	453	(60.5)
	**Age at diagnosis**	Mean (SD)	56.4 (14.7)	
	**Age group**	≤39	119	(15.9)
		40–49	95	(12.7)
		50–59	165	(22.0)
		60–69	225	(30.0)
		≥70	145	(19.4)
	**Year of diagnosis**	2015	151	(20.2)
		2016	136	(18.2)
		2017	163	(218)
		2018	147	(19.6)
		2019	152	(20.3)
	**Ethnic group**	White British	484	(64.6)
		Asian/Asian British	11	(1.5)
		Black/Black British	23	(3.1)
		Mixed Ethnic Group	10	(1.3)
		Any Other White	30	(4.1)
		Any Other Ethnic Group	19	(2.5)
		Not Stated/Specified	172	(23.0)
	**Performance status at referral**	0	312	(41.7)
		1	244	(32.6)
		2	63	(8.4)
		3/4	27	(3.6)
		Unknown	103	(13.8)
	**Vital status**	Alive	210	(28.0)
	(on 30 July 2021)	Dead	539	(72.0)
	**Histology**	Glioblastoma	570	(76.1)
**Tumour characteristics**		Astrocytoma	98	(13.1)
		Oligodendroglioma	81	(10.8)
	**WHO grade**	I/II	77	(10.3)
		III/IV	672	(89.7)
	**Tumour location**	Frontal Lobe	237	(31.6)
		Occipital Lobe	27	(3.6)
		Parietal Lobe	116	(15.5)
		Temporal Lobe	176	(23.5)
		Overlapping Lesion of Brain	131	(17.5)
		Cerebrum	31	(4.1)
		Ventricle	10	(1.3)
		Brain, other	21	(2.8)
	**IDH1 mutation**	Mutated	146	(19.5)
		Wild-type	587	(78.4)
		Not Tested/Unknown	16	(2.1)
	**MGMT promoter methylation**	Methylated	407	(54.3)
		Unmethylated	291	(38.9)
		Not Tested/Unknown	51	(6.8)
	**1p19q co-deletion**	Co-deleted	86	(11.5)
		Not Co-deleted	50	(6.7)
		Not Tested/Unknown	613	(81.8)
	**Resection type**	Total/Total Macroscopic	149	(19.9)
		Gross Subtotal/Partial	192	(25.6)
		Extent Uncertain	408	(54.5)

**Table 2:** Frequency of molecular biomarkers by tumour histology for 749 glioma cases diagnosed during 2015–2019.

	Glioblastoma N = 570 (76.1%)		Astrocytoma N = 98 (13.1%)		Oligodendroglioma N = 81 (10.8%)		Total		*p*-Value
**IDH Mutation**									
IDH1Mutated	19	(3.3%)	52	(53.1%)	75	(92.6%)	146	(19.5%)	*<0.001*
Wildtype	550	(96.5%)	31	(31.6%)	6	(7.4%)	587	(78.4%)	
Not Tested/Unknown	1	(0.2%)	15	(15.3%)	0	(0.0%)	16	(2.1%)	
**MGMT Promoter Methylation**									
MGMT methylated	285	(50.0%)	60	(61.2%)	62	(76.5%)	407	(54.3%)	*<0.001*
Unmethylated	277	(48.6%)	12	(12.2%)	2	(2.5%)	291	(38.9%)	
Not Tested/Unknown	8	(1.4%)	26	(26.5%)	17	(21%)	51	(6.8%)	
**1p19q Co-deletion**									
Co-deleted	6	(1.0%)	4	(4.1%)	76	(93.8%)	86	(11.5%)	*<0.001*
Not Co-deleted	21	(3.7%)	24	(24.5%)	5	(6.2%)	50	(6.7%)	
Not Tested/Unknown	543	(95.3%)	70	(71.4%)	0	(0.0%)	613	(81.8%)	

**Table 3:** Odds ratios for IDH1 mutation in glioma cancer patients diagnosed during 2015–2019.

	Unadjusted			Adjusted for Age, Sex, PS, Ethnicity			and Tumour Biomarkers			and Tumour Histology, Tumour Location			and Extent of Resection		
	OR	95% CI		OR	95% CI		OR	95% CI		OR	95% CI		OR	95% CI	
**Age at diagnosis**															
≤39	5.48	3.17	9.47	4.86	2.77	8.54	6.04	2.48	14.73	2.18	0.75	6.39	1.83	0.61	5.50
40–49	2.45	1.35	4.42	2.34	1.27	4.30	3.78	1.42	10.08	2.63	0.80	8.60	2.29	0.70	7.51
50–59	1.00			1.00			1.00			1.00			1.00		
60–69	0.45	0.25	0.84	0.48	0.26	0.89	0.53	0.21	1.37	0.60	0.18	1.95	0.51	0.15	1.72
≥70	0.20	0.08	0.51	0.20	0.08	0.51	0.46	0.14	1.53	0.61	0.16	2.38	0.65	0.16	2.60
*χ2 and p-value*	*χ2 (4) = 103.85; p < 0.001*			*χ2 (4) = 86.56; p < 0.001*			*χ2 (4) = 40.42; p < 0.001*			*χ2 (4) = 9.03; p = 0.0603*			*χ2 (4) = 7.54; p = 0.1098*		
**Sex**															
Male	1.00			1.00			1.00			1.00			1.00		
Female	1.11	0.76	1.60	0.99	0.65	1.50	0.66	0.35	1.24	0.72	0.33	1.55	0.72	0.33	1.58
*χ2 and p-value*	*χ2 (1) = 0.29; p = 0.5913*			*χ2 (1) = 0.00; p = 0.9456*			*χ2 (1) = 1.65; p = 0.1984*			*χ2 (1) = 0.70; p = 0.4012*			*χ2 (1) = 0.65; p = 0.4193*		
**Ethnic groups**															
White British	1.00			1.00			1.00			1.00			1.00		
Any Other Ethnic Group	1.70	0.63	4.58	1.08	0.36	3.27	1.50	0.36	6.19	3.00	0.57	15.63	2.61	0.50	13.58
Any Other White	1.84	0.84	4.06	1.43	0.58	3.54	1.75	0.39	7.90	1.23	0.18	8.60	1.38	0.20	9.50
Asian/Asian British	3.68	1.05	12.97	2.30	0.49	10.78	1.56	0.13	18.47	1.20	0.03	54.51	1.11	0.03	39.17
Black/Black British	0.55	0.16	1.90	0.42	0.11	1.64	0.93	0.17	4.96	0.65	0.08	5.17	0.68	0.09	5.39
Mixed Ethnic Group	0.41	0.05	3.27	0.17	0.02	1.47	0.03	0.00	0.55	0.11	0.00	2.64	0.14	0.01	2.95
Not Stated/Specified	0.50	0.30	0.83	0.57	0.33	1.01	0.59	0.26	1.33	0.60	0.22	1.61	0.59	0.22	1.60
*χ2 and p-value*	*χ2 (6) = 17.87; p = 0.0066*			*χ2 (6) = 9.90; p = 0.1288*			*χ2 (6) = 8.34; p = 0.2140*			*χ2 (6) = 5.02; p = 0.5414*			*χ2 (6) = 4.46; p = 0.6148*		
**MGMT methylation status**															
Unmethylated	1.00			1.00			1.00			1.00			1.00		
Methylated	15.92	7.30	34.75	19.73	8.64	45.08	18.74	6.31	55.68	13.33	3.78	47.08	14.13	3.88	51.43
*χ2 and p-value*	*χ2 (1) = 48.33; p < 0.001*			*χ2 (1) = 50.18; p < 0.001*			*χ2 (1) = 27.56; p < 0.001*			*χ2 (1) = 16.31; p = 0.0001*			*χ2 (1) = 16.21; p = 0.0001*		
**Tumour location**															
Frontal	1.00			1.00			1.00			1.00			1.00		
Brain, other	0.54	0.17	1.68	0.30	0.09	1.03	0.64	0.07	5.52	1.32	0.14	12.52	2.30	0.22	23.84
Cerebrum	0.08	0.01	0.58	0.05	0.01	0.40	0.11	0.01	1.26	0.06	0.00	0.85	0.08	0.00	1.82
Occipital Lobe	0.17	0.04	0.73	0.16	0.03	0.77	0.26	0.04	1.65	0.33	0.03	3.19	1.02	0.10	10.57
Overlapping Brain Lesions	0.48	0.29	0.81	0.68	0.37	1.23	1.12	0.47	2.67	1.33	0.47	3.74	1.69	0.56	5.08
Parietal Lobe	0.28	0.15	0.52	0.38	0.19	0.77	0.53	0.19	1.46	0.50	0.15	1.65	0.62	0.17	2.25
Temporal Lobe	0.28	0.17	0.48	0.35	0.19	0.63	0.44	0.18	1.06	0.41	0.15	1.17	0.67	0.23	1.99
Ventricle	0.29	0.03	2.39	0.20	0.02	1.92	0.16	0.01	3.57	0.09	0.00	1.83	0.08	0.00	2.19
*χ2 and p-value*	*χ2 (7) = 39.34; p < 0.001*			*χ2 (7) = 27.16; p = 0.0003*			*χ2 (7) = 9.60; p = 0.2122*			*χ2 (7) = 11.04; p = 0.1370*			*χ2 (7) = 7.84; p = 0.3466*		
**Tumour histology**															
Astrocytoma	1.00			1.00			1.00			1.00			1.00		
Glioblastoma	0.02	0.01	0.04	0.03	0.01	0.07	0.05	0.02	0.12	0.04	0.02	0.11	0.03	0.01	0.09
Oligodendroglioma	7.45	2.90	19.13	10.54	3.73	29.78	2.49	0.40	15.56	2.47	0.37	16.61	3.73	0.47	29.79
*χ2 and p-value*	*χ2 (2) = 215.81; p < 0.001*			*χ2 (2) = 154.87; p < 0.001*			*χ2 (2) = 55.46; p < 0.001*			*χ2 (2) = 55.89; p < 0.001*			*χ2 (2) = 53.13; p < 0.001*		
**Extent of resection**															
Extent Uncertain	1.00			1.00			1.00			1.00			1.00		
Gross Total/Total Macroscopic	1.95	1.24	3.08	1.87	1.11	3.17	1.92	0.88	4.20	2.73	1.01	7.33	2.73	1.01	7.33
Subtotal/Partial	1.13	0.71	1.80	1.33	0.78	2.26	1.09	0.49	2.42	1.77	0.63	5.00	1.77	0.63	5.00
*χ2 and p-value*	*χ2 (2) = 8.59; p = 0.0136*			*χ2 (2) = 5.50; p = 0.0638*			*χ2 (2) = 2.86; p = 0.2387*			*χ2 (2) = 4.08; p = 0.1299*			*χ2 (2) = 4.08; p = 0.1299*		

Abbreviations: OR = Odds ratio, CI = Confidence interval, PS = Performance status.

**Table 4:** Odds ratios for MGMT methylated promoter in glioma cancer patients diagnosed during 2015–2019.

	Unadjusted			Adjusted for Age, Sex, PS, Ethnicity			and Tumour Biomarkers			and Tumour Histology, Tumour Location			and Extent of Resection		
	OR	95% CI		OR	95% CI		OR	95% CI		OR	95% CI		OR	95% CI	
**Age at diagnosis**															
≤39	2.56	1.48	4.43	2.54	1.45	4.45	1.42	0.73	2.77	1.15	0.54	2.45	1.20	0.56	2.56
40–49	1.32	0.77	2.27	1.29	0.75	2.24	0.83	0.44	1.57	0.82	0.42	1.57	0.82	0.43	1.59
50–59	1.00			1.00			1.00			1.00			1.00		
60–69	1.35	0.90	2.05	1.45	0.95	2.21	1.69	1.08	2.67	1.73	1.09	2.73	1.76	1.11	2.78
≥70	1.11	0.70	1.75	1.18	0.74	1.88	1.56	0.95	2.55	1.56	0.95	2.57	1.53	0.93	2.53
*χ2 and p-value*	*χ2 (4) = 12.41; p = 0.0145*			*χ2 (4) = 11.42; p = 0.0223*			*χ2 (4) = 9.01; p = 0.0608*			*χ2 (4) = 9.26; p = 0.0550*			*χ2 (4) = 9.28; p = 0.0544*		
**Sex**															
Male	1.00			1.00			1.00			1.00			1.00		
Female	1.52	1.11	2.08	1.54	1.11	2.12	1.72	1.22	2.44	1.72	1.20	2.46	1.75	1.22	2.51
*χ2 and p-value*	*χ2 (1) = 6.76; p = 0.0093*			*χ2 (1)= 6.77; p = 0.0093*			*χ2 (1) = 9.39; p = 0.0022*			*χ2 (1) = 8.88; p = 0.0029*			*χ2 (1) = 9.38; p = 0.0022*		
**Ethnic groups**															
White British	1.00			1.00			1.00			1.00			1.00		
Any Other Ethnic Group	0.94	0.37	2.39	0.78	0.30	2.03	0.82	0.27	2.48	0.90	0.29	2.76	0.92	0.30	2.82
Any Other White	0.86	0.39	1.87	0.73	0.33	1.63	0.59	0.24	1.48	0.52	0.20	1.36	0.49	0.19	1.28
Asian/Asian British	0.69	0.17	2.78	0.62	0.14	2.66	0.38	0.06	2.37	0.47	0.07	2.90	0.44	0.07	2.78
Black/Black British	0.69	0.28	1.68	0.69	0.27	1.74	0.79	0.30	2.10	0.76	0.28	2.04	0.75	0.28	2.01
Mixed Ethnic Group	1.60	0.41	6.27	1.17	0.29	4.76	1.53	0.36	6.50	1.36	0.32	5.84	1.37	0.32	5.90
Not Stated/Specified	0.90	0.63	1.29	0.93	0.64	1.35	1.01	0.68	1.50	1.00	0.67	1.50	1.00	0.67	1.48
*χ2 and p-value*	*χ2 (6) = 1.79; p = 0.9379*			*χ2 (6) = 1.80; p = 0.9373*			*χ2 (6) = 3.01; p = 0.8070*			*χ2 (6) = 2.95; p = 0.8156*			*χ2 (6) = 3.36; p = 0.7631*		
**IDH1 mutation**															
Unmutated	1.00			1.00			1.00			1.00			1.00		
Mutated	15.92	7.30	34.75	19.88	8.72	45.33	16.71	6.15	45.43	12.61	4.14	38.41	15.54	4.73	51.05
*χ2 and p-value*	*χ2 (1) = 48.33; p < 0.001*			*χ2 (1) = 50.56; p < 0.001*			*χ2 (1) = 30.46; p < 0.001*			*χ2 (1) = 19.88; p < 0.001*			*χ2 (1) = 20.42; p < 0.001*		
**Tumour location**															
Frontal	1.00			1.00			1.00			1.00			1.00		
Brain, other	0.46	0.17	1.23	0.38	0.14	1.07	0.62	0.21	1.82	0.65	0.22	1.94	0.60	0.20	1.82
Cerebrum	0.34	0.15	0.80	0.28	0.12	0.67	0.43	0.17	1.07	0.43	0.17	1.07	0.40	0.16	1.01
Occipital Lobe	0.61	0.26	1.42	0.66	0.27	1.62	0.88	0.34	2.27	0.86	0.33	2.25	0.75	0.28	2.02
Overlapping Brain Lesions	0.81	0.52	1.28	0.83	0.51	1.33	0.93	0.56	1.55	0.95	0.57	1.58	0.93	0.55	1.58
Parietal Lobe	0.62	0.39	0.98	0.67	0.41	1.08	0.88	0.53	1.48	0.90	0.53	1.52	0.87	0.51	1.47
Temporal Lobe	0.55	0.36	0.82	0.56	0.37	0.87	0.71	0.45	1.13	0.71	0.45	1.13	0.68	0.42	1.09
Ventricle	3.08	0.36	26.06	1.80	0.20	15.93	3.40	0.38	30.69	3.08	0.34	27.96	3.03	0.33	27.55
*χ2 and p-value*	*χ2 (7) = 15.90; p = 0.0261*			*χ2 (7) = 15.37; p = 0.0316*			*χ2 (7) = 6.80; p = 0.4499*			*χ2 (7) = 6.59; p = 0.4732*			*χ2 (7) = 7.48; p = 0.3805*		
**Tumour histology**															
Astrocytoma	1.00			1.00			1.00			1.00			1.00		
Glioblastoma	0.21	0.11	0.39	0.19	0.09	0.39	0.45	0.19	1.08	0.46	0.19	1.12	0.44	0.18	1.09
Oligodendroglioma	6.20	1.33	28.88	7.01	1.45	33.91	0.21	0.02	2.45	0.22	0.02	2.51	0.10	0.01	1.80
*χ2 and p-value*	*χ2 (2) = 44.17; p < 0.001*			*χ2 (2) = 40.62; p < 0.001*			*χ2 (2) = 4.34; p = 0.1141*			*χ2 (2) = 4.05; p = 0.1318*			*χ2 (2) = 5.03; p = 0.0808*		
**Extent of resection**															
Extent Uncertain	1.00			1.00			1.00			1.00			1.00		
Gross Total/Total Macroscopic	0.95	0.64	1.40	0.84	0.56	1.26	0.70	0.44	1.10	0.68	0.43	1.08	0.68	0.43	1.08
Subtotal/Partial	0.95	0.66	1.37	0.97	0.66	1.41	0.93	0.62	1.40	0.93	0.61	1.40	0.93	0.61	1.40
*χ2 and p-value*	*χ2 (2) = 0.11; p = 0.9450*			*χ2 (2) = 0.72; p = 0.6994*			*χ2 (2) = 2.47; p = 0.2903*			*χ2 (2) = 0.11; p = 0.9450*			*χ2 (2) = 0.11; p = 0.9450*		

Abbreviations: OR = Odds ratio, CI = Confidence interval, PS = Performance status.

## Data Availability

The data are collated, maintained, and quality assured by the Neurosciences Department within King’s College Hospital NHS Foundation Trust.
